# Cross-country comparison of victimisation-related injury admission in children and adolescents in England and Western Australia

**DOI:** 10.1186/1472-6963-13-260

**Published:** 2013-07-06

**Authors:** Arturo Gonzalez-Izquierdo, Allison Ward, Melissa O’Donnell, Leah Li, Andreas Roposch, Fiona Stanley, Ruth Gilbert

**Affiliations:** 1 MRC Centre of Epidemiology for Child Health, UCL Institute of Child Health, 30 Guilford Street, London WC1N 1EH, UK; 2 General and Adolescent Paediatrics Unit, UCL Institute of Child Health, London WC1N 1EH, UK; 3 Telethon Institute for Child Health Research, Centre for Child Health Research, University of Western Australia, Perth 6008, Western Australia, Australia; 4 Department of Surgery, UCL Institute of Child Health, London WC1N 1EH, UK

**Keywords:** Victimisation, Children, Adolescents, Injuries, ICD-10 codes

## Abstract

**Background:**

A single, standardised measure of victimisation-related (VR) injury admission in hospital administrative datasets could allow monitoring of preventive and response strategies and international comparisons of policy. Consistency of risk factors and incidence rates for a measure of victimisation-related injury in different countries with similar access to healthcare services would provide indirect evidence for measure validity.

**Methods:**

Cohorts were derived from hospital administrative data for children aged less than 18 years who were admitted for acute injury to hospitals in England or Western Australia (WA) in 2000 to 2008. We compared the effects of age, sex and deprivation on the annual incidence of acute admission for VR injury defined by a cluster of ICD-10 codes reflecting characteristics that should alert clinicians to consider victimisation as a cause of injury. Four subcategories comprised codes specifically indicating child maltreatment, assault, undetermined cause, or adverse social circumstances.

**Results:**

The incidence of VR injury followed a similar ‘J’-shaped association with age in both countries with increasing rates from 10 years onwards and peaks in infancy and in 16–17 year-olds. In both countries, rates increased with deprivation. Girls had lower rates than boys except in the 11–15 age group where girls had higher rates than boys in WA but not in England. Adjusted incidence rates were similar in both countries for children aged 3 to 15 years old, but were higher in WA compared with England in children under 3 years old and in those aged 16–17 years. Higher rates in WA in 16–17 year-olds were explained by more admissions coded for the subcategories of adverse social circumstances, and to a lesser extent, assault, than in England. Children less than 3 years old were more often coded specifically for maltreatment in WA than in England.

**Conclusions:**

The similarities in risk factors and in the adjusted rates of victimisation-related injury admission in both countries suggest that the VR cluster of ICD-10 codes is measuring a similar underlying problem. Differential use of coding subcategories highlights the need to use the entire VR cluster for comparisons across settings.

## Background

Childhood victimisation is the term given to any adversity or harm suffered by children or adolescents as a result of the actions of others. The term victimisation encompasses child maltreatment (physical abuse, sexual abuse, emotional abuse or neglect by carers), conventional crime (assault and theft), peer and sibling victimisation (youth violence and bullying), and indirect victimisation (the witnessing of domestic or community violence) [1].

Evidence from community surveys shows that victimisation is common, it occurs across the entire childhood age range, affects many of the same children and young people, and has similarly serious adverse outcomes regardless of whether the perpetrator was the carer or another individual [2-6]. There is growing recognition among policy makers that the preventive and protective responses to child maltreatment need to be broadened to address the full range of victimisation [6,7]. To do this, policy makers need information on the frequency and patterns of presentations to services across the entire age range. Data from statutory services provide a fragmented picture as youth violence tends to report to police and child maltreatment to child protection services. However, victimised children and young people who sustain severe injury are likely to present to healthcare regardless of the perpetrator. Children and adolescents who are victimised may present to primary care, emergency departments or as admissions to hospital [8-10]. Of these data sources, only administrative data for hospital admissions provides sufficiently detailed and standardised information to assess rates and risk factors for injury related to victimisation in cross-country comparisons [10-13].

We used guidelines by the National Institute for Health and Care Excellence (NICE) to develop a measure of VR injury admission based on ICD-10 codes that reflect features for possible maltreatment or other forms of victimisation that should warrant further action by clinicians [10,12,14]. As a first step to validating this measure, we assessed variation in risk factors and incidence rates for VR injury admission and its four constituent coding clusters – indicating child maltreatment, assault, undetermined cause, or adverse social circumstances – in England and WA [10,12]. We hypothesised similar patterns in both countries as they both have universal health care systems with primary care as the referral pathway for secondary services; they have well-established, population-based healthcare data, and similar cultural and economic risk factors for childhood victimisation (e.g., alcohol consumption, child poverty, child health and other social inequalities) [10].

## Methods

### Study population

We conducted a retrospective cohort study using hospital administrative data for all childhood injury admissions to the NHS in England (Hospital Episode Statistics) and to all hospitals in WA (Hospital Morbidity Data System) over a 9 year period (2000 to 2008) [15]. Data sources and coding systems have been reported elsewhere [12,15].

We defined cohorts of children under 18 years of age who were acutely admitted to hospital with at least one ICD-10 code in their discharge data that reflected injury or poisoning (all S and T codes) [16]. We excluded injury admissions that could be related to birth by excluding first week admissions in England. In WA, data providers did not make admissions in the first week separately identifiable so we excluded birth-related admissions (with ICD-10 code Z38) instead. In both cohorts, any re-admission within two days of discharge was counted as the same admission. Recurrent acute admissions in the same child and calendar year were negligible and were counted as separate admissions.

### Outcome

Because of the well-documented under-ascertainment of victimisation-related events in hospital administrative data, [17-21] we used a range of ICD-10 codes for maltreatment, assault and other alert features for ‘considering’ child maltreatment in the NICE guidelines on when to suspect child maltreatment [14]. Codes were classified into an exclusive, descending hierarchy of four groups comprising: maltreatment syndrome, assault, undetermined intent and codes reflecting adverse social circumstances (Additional file 1: Table S1) [12]. Maltreatment syndrome or assault denote explicit reference to maltreatment or inflicted injury. These codes are likely to be highly specific for victimisation but lack sensitivity because staff may be reluctant to use such pejorative labels if the cause of injury is uncertain, as is often the case because investigations take time to complete [12,20]. The groups of codes for undetermined intent or for alert features about the child’s family environment or care – referred to as adverse social circumstances related to the injury [10,12] – were included to capture less certain cases where clinician concerns had been raised. These four clusters were initially developed to identify VR injury admissions in England and then used to identify the same type of admissions in WA.

### Risk factors

We analysed risk factors that were routinely available in hospital administrative data (age in years, sex and deprivation quintile). Deprivation quintile was derived from the Index of Relative Social Disadvantage for WA and from the Index of Multiple Deprivation for England [22,23]. Because of the strong relationship between age and incidence of victimisation-related injury, all analyses were stratified into four age groups (<3; 3–10; 11–15 and 16–17 years). These age groups were chosen to produce adequate numbers in each stratum and to reflect child development and decreasing levels of supervision as the child moves from nursery through school settings and to reflect the shift from paediatric to adult hospital services at 16 years. Patterns of victimisation-related admissions and the effects of risk factors were similar for narrower age bands within these groups (results available from authors).

### Analyses

We calculated crude incidence rates for VR injury admission overall and separately for the four sub-categories, using the estimated mid-year population as the denominator (Table 1) [24,25]. We also derived the prevalence of VR injury as a proportion of all acute injury admissions to provide a relevant measure for healthcare providers.

**Table 1:** **Incidence and prevalence of victimisation**^**1 **^**in children aged 0–17 years admitted to hospital with injury**

**Age group (years)**	**Cause of injury**^**2**^	**Incidence**				**Prevalence**			
		**England**		**Western Australia**		**England**		**Western Australia**	
		**IR**^**3**^	**95%CI**	**IR**	**95%CI**	**n**	**(%)**	**n**	**(%)**
<3	Victimisation^4^	51.6	(50.5, 52.7)	108.0	(100.3, 115.7)	8,243	(3.5)	755	(5.7)
	Maltreatment	16.9	(16.3, 17.6)	61.5	(55.7, 67.3)	2,705	(1.1)	430	(3.3)
	Assault	5.3	(4.9, 5.7)	8.2	(6.0, 10.3)	846	(0.4)	57	(0.4)
	Undetermined intent	14.9	(14.3, 15.5)	9.9	(7.5, 12.2)	2,380	(1.0)	69	(0.5)
	Adverse social circumstances	14.5	(13.9, 15.1)	28.5	(24.5, 32.4)	2,312	(1.0)	199	(1.5)
	**Total injury admissions**	**1,487.8**	**(1481.8, 1493.8)**	**1,884.5**	**(1852.3, 1916.6)**	**237,766**	**(100)**	**13179**	**(100)**
	Mid-year population estimate	15,981,100		699354					
3 - 10	Victimisation	15.4	(15, 15.8)	22.4	(20.3, 24.5)	6,666	(1.5)	434	(1.7)
	Maltreatment	2.8	(2.7, 3)	6.2	(5.1, 7.3)	1,219	(0.3)	120	(0.5)
	Assault	4.1	(3.9, 4.3)	5.6	(4.6, 6.7)	1,766	(0.4)	109	(0.4)
	Undetermined intent	5.1	(4.9, 5.3)	2.9	(2.2, 3.7)	2,192	(0.5)	57	(0.2)
	Adverse social circumstances	3.4	(3.3, 3.6)	7.6	(6.4, 8.9)	1,489	(0.3)	148	(0.6)
	**Total injury admissions**	**1,002.7**	**(999.7, 1005.7)**	**1,342.7**	**(1326.4, 1359)**	**433,626**	**(100)**	**26051**	**(100)**
	Mid-year population estimate	43,245,100		1940156					
11 - 15	Victimisation	95.6	(94.4, 96.7)	124.6	(118.5, 130.7)	27,517	(7.2)	1603	(8.3)
	Maltreatment	3.2	(3, 3.4)	5.8	(4.4, 7.1)	912	(0.2)	74	(0.4)
	Assault	47.4	(46.6, 48.2)	46.8	(43.1, 50.5)	13,640	(3.5)	602	(3.1)
	Undetermined intent	11.2	(10.8, 11.6)	14.8	(12.7, 16.9)	3,224	(0.8)	190	(1.0)
	Adverse social circumstances	33.8	(33.2, 34.5)	57.3	(53.2, 61.4)	9,741	(2.5)	737	(3.8)
	**Total injury admissions**	**1,336.1**	**(1331.9, 1340.4)**	**1,503.9**	**(1482.7, 1525.1)**	**384,759**	**(100)**	**19343**	**(100)**
	Mid-year population estimate	28,796,400		1286214					
16 - 17	Victimisation	195.7	(193.1, 198.2)	448.3	(430.1, 466.5)	22,713	(13.6)	2333	(23.4)
	Maltreatment	1.2	(1, 1.4)	8.6	(6.1, 11.2)	135	(0.1)	45	(0.5)
	Assault	143.7	(141.5, 145.9)	224.2	(211.4, 237.1)	16,677	(10.0)	1167	(11.7)
	Undetermined intent	13.1	(12.4, 13.8)	34.8	(29.7, 39.8)	1,521	(0.9)	181	(1.8)
	Adverse social circumstances	37.7	(36.6, 38.9)	180.6	(169.1, 192.2)	4,380	(2.6)	940	(9.4)
	**Total injury admissions**	**1,437.4**	**(1430.5, 1444.3)**	**1,918.2**	**(1880.6, 1955.9)**	**166,843**	**(100)**	**9983**	**(100)**
	Mid-year population estimate	11,607,100		520423					

^1^ Recorded victimisation in England and Western Australia between 2000 – 2008, inclusive, according to age and cause of injury.

^2^ Descending, exclusive hierarchy.

^3^ Incidence rate per 100,000.

^4^ Victimisation includes maltreatment, assault, undetermined intent and adverse social circumstances.

We restricted analyses of risk factors to the overall VR cluster of codes and did not explore effects on sub-categories due to small cell sizes and to avoid multiple testing. We used negative binomial regression models (a generalisation of Poisson regression models) to estimate the incidence rate ratios for country (WA vs. England), age, sex (female vs. male), and deprivation quintile (vs. least deprived) taking into account over-dispersion of the data (Table 2).

**Table 2:** Risk factors for victimisation-related injury admission: multivariable analyses (incidence rate ratios and 95% confidence limits)

**Age group (years)**	**Factors**	**Multivariable (no interactions)**	
		**IRR**	**(95%CI)**
<3y			
	Western Australia	1.35	(1.22, 1.50)
	Year of age	0.6	(0.58, 0.63)
	Female	0.8	(0.75, 0.86)
	2^nd^ least deprived	1.04	(0.91, 1.19)
	3	1.65	(1.46, 1.86)
	4	2.74	(2.44, 3.08)
	Most deprived	5.82	(5.2, 6.52)
3 – 10			
	Western Australia	0.99	(0.86, 1.14)
	Year of age	0.96	(0.94, 0.98)
	Female	0.62	(0.57, 0.69)
	2^nd^ least deprived	1.09	(0.92, 1.29)
	3	1.58	(1.34, 1.86)
	4	2.61	(2.23, 3.06)
	Most deprived	4.84	(4.15, 5.63)
11 – 15			
	Western Australia	0.92	(0.82, 1.03)
	Year of age	1.72	(1.65, 1.79)
	Female	1.01	(0.91, 1.13)
	2^nd^ least deprived	1.06	(0.88, 1.26)
	3	1.42	(1.19, 1.7)
	4	1.98	(1.66, 2.35)
	Most deprived	3.28	(2.77, 3.89)
16 – 17			
	Western Australia	1.84	(1.57, 2.15)
	Year of age	1.14	(0.98, 1.33)
	Female	0.53	(0.45, 0.62)
	2^nd^ least deprived	0.94	(0.74, 1.21)
	3	1.26	(0.99, 1.61)
	4	1.6	(1.25, 2.03)
	Most deprived	2.58	(2.03, 3.28)

IRR = incidence rate ratio.

Overall p-value <0.001 for all models.

The multiple regression models included all covariates. We used the Akaike Information Criteria (AIC) to select the model with the best goodness of fit [26]. A p-value of less than 0.05 was regarded as statistically significant. To assess whether the effect of risk factors differed in WA compared with England, we tested for the interaction between country and age, sex, and deprivation quintile (as a continuous measure for interaction models) for each age group. Results are reported separately for risk factors which had a significant interaction with country (Table 3).

**Table 3:** Incidence rate ratios for victimisation-related injury admission: multivariable analyses where interaction with country was significant

**Age group**	**Factor**	**England**	**95%CI**	**Western Australia**	**95%CI**	p-value for interaction w/country**
11 – 15*	Female	0.83	(0.74, 0.95)	1.5	(1.25, 1.79)	<0.001
16 – 17*	Deprivation	1.32	(1.25, 1.39)	1.21	(1.15, 1.28)	0.04
	Female	0.37	(0.32, 0.44)	0.83	(0.71, 0.98)	<0.001

* Adjusted by year of age and deprivation.

** log (μ) = a + b. WA + c. Deprivation + d. WA. Deprivation + e. Age + f. WA. Age + g. Gender + h. WA. Gender.

Where a, c, e and g are the intercept and coefficients for England, and b, d, f and h are the intercept and coefficients for Western Australia. Deprivation used as numeric (Incidence rate ratio represents increased risk per quintile of deprivation, from least to most deprived).

### Sensitivity analyses

To address the possibility that risk factors for victimisation-related injury admission might be dominated by certain types of injury (e.g. ingestion/poisoning) in some groups, we repeated the analyses for head injury (codes S00 – S09), which is strongly associated with child victimisation [27,28].

### Ethics

Permissions to use the anonymised datasets were granted by the relevant data custodians in England and WA. In WA ethics approval was obtained from the University of Western Australia Human Ethics Committee, Department of Health Human Research Ethics Committee and the Western Australian Aboriginal Human Information and Ethics Committee. Use of anonymised data in England for the purpose in this study did not require research ethics approval but met the requirements of the data provider [29].

## Results

### Crude rates

**Figure 1:**
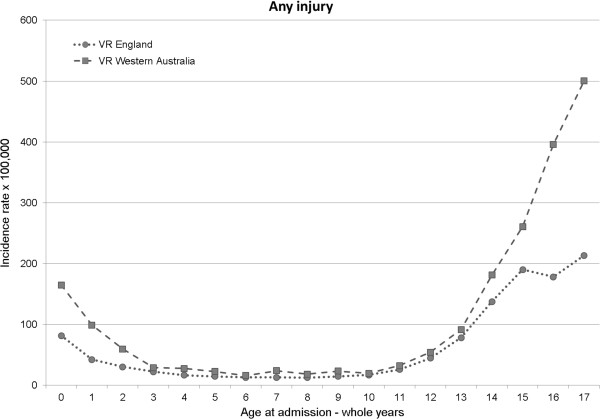
Incidence of victimisation-related injury admission according to year of age in England and Western Australia.

There was a J’-shaped relationship between the incidence of VR injury admission and year of age in both countries. Crude incidence rates were higher in WA in all age groups compared with England, particularly at the extremes of age (i.e. early years 0–3 years and adolescence >13 years – Table 1, Figure 1). The prevalence of victimisation-related injury admission as a proportion of all acute injury admissions was highest in 16 to 17 year olds (13.6% in England and 23.4% in WA, Table 1).

**Figure 2:**
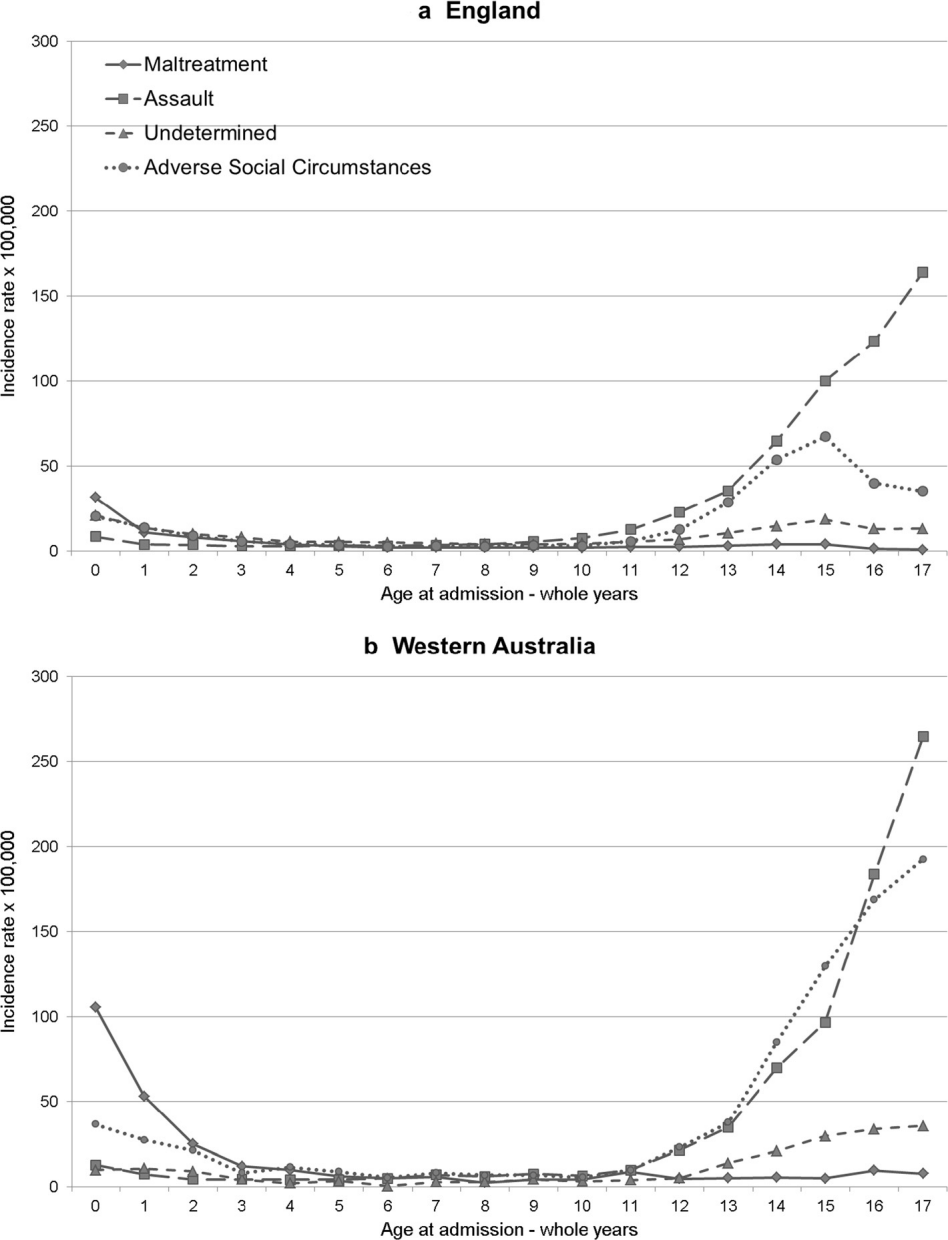
Incidence of victimisation-related injury admission according to age and coding sub-group (2000 – 2008) in (a) England and (b) Western Australia.

Differences in crude incidence rates were partly explained by higher rates of total injury admission in WA compared with England (Table 1). There were also striking differences in the use of the coding subcategories at the extremes of age (Figure 2a and b; Table 1). In 16–17 year olds, there were 142.9/100,000 more admissions coded for adverse social circumstances in WA than in England, and 80.5/100,000 more coded for assault. However, the proportion of acute injury admissions that were coded for assault was similar in both countries, whereas a much greater proportion in WA was coded for adverse social circumstance (Table 1 and Figure 2b). Among children less than 3 years old, there were 44.6/100,000 more admissions specifically coded for maltreatment in WA compared with England and these cases made up a greater proportion of total injury admissions in WA.

### Effects of risk factors

The results of the multiple regression model showed that after adjusting for risk factors, there were no significant differences in the incidence of VR injury admission in WA compared with England for the middle age ranges (3 to 15 years), but rates were higher in WA at the extremes of age. Multiple regression models showed significantly higher incidence rates of VR injury admission in WA than in England for children less than 3 years (35% higher) and adolescents aged 16–17 years (84% higher; Table 2). Higher levels of social deprivation were associated with increasing rates of victimisation-related injury admission in both countries (Table 2), although an interaction with country that was of borderline significance (p = 0.04) for 16–17 year-olds, suggests that this effect may have been stronger in England than in WA for this age group.

**Figure 3:**
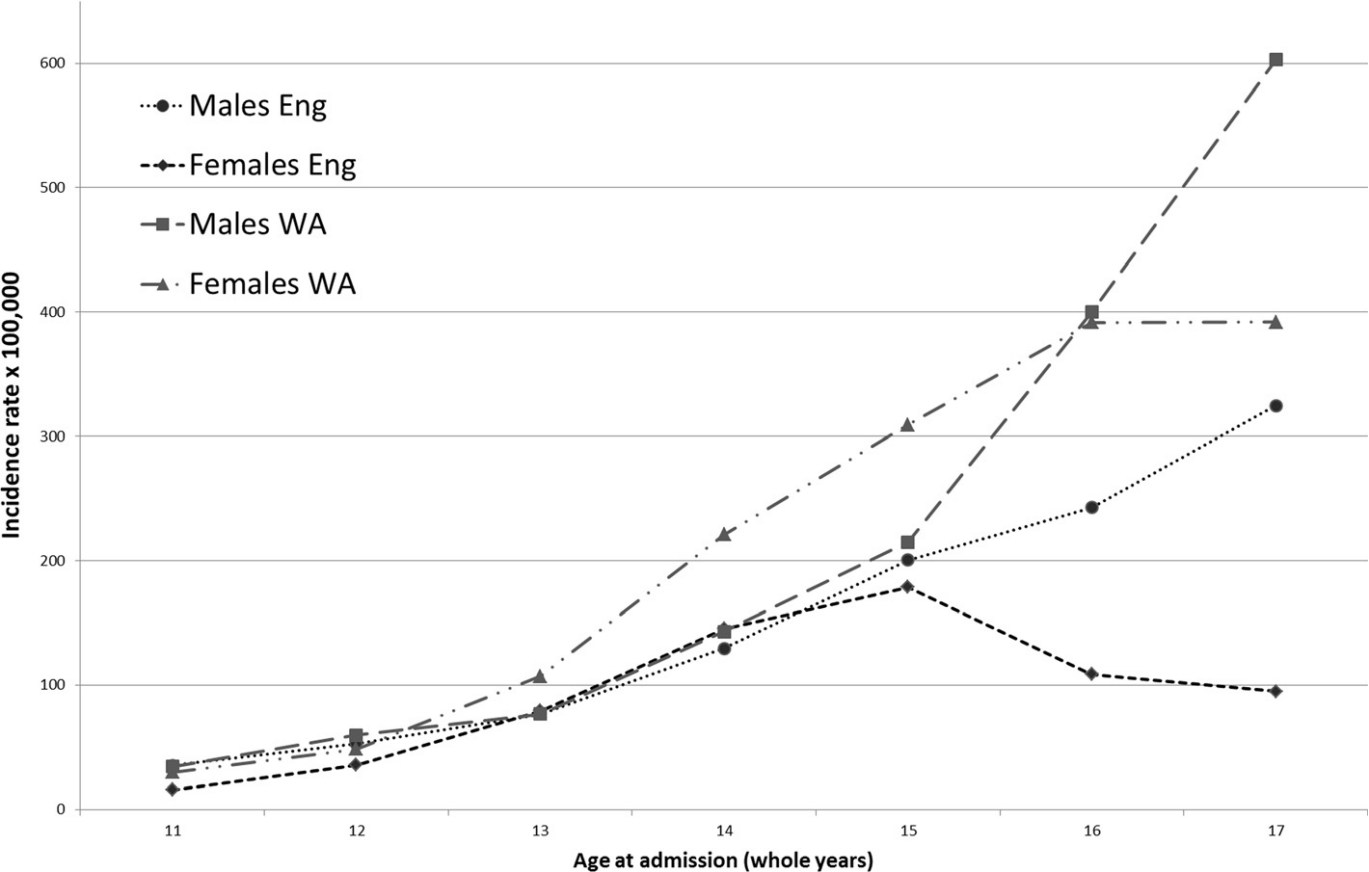
Incidence of victimisation-related injury admission in adolescence by gender in England and Western Australia.

There were fewer VR injury admissions in girls than boys in both countries in children less than 11 years old. In adolescents aged 11 to 15 year, the gender association with VR differed between the two countries (p-value < 0.001, Table 3). Girls had lower rates than boys in England, but significantly higher rates than boys in WA (Table 3 and Figure 3). In 16 to 17 year olds, girls in both countries had lower rates than boys, but this difference was attenuated in WA (Table 3 and Figure 3). Higher rates of victimisation-related injury in WA compared with England in both sexes were largely explained by codes for adverse social circumstances and assault (Additional file 2: Figure S1).

### Sensitivity analyses

Head injury admissions comprised 27% of all acute injury admissions in both countries (Additional file 1: Table S2). All differences between countries in crude and adjusted incidence rates and in the effects of risk factors were attenuated in sensitivity analyses confined to head injuries. Adjusted incidence rates of VR head injury admission were similar for children less than 11 years old, lower in WA compared with England for 11 to 15 year olds, and higher in WA compared with England for 16 to 17 year olds (Additional file 1: Table S3). Girls of all ages were less likely to be admitted for VR head injury than boys, but this sex difference was reduced in WA for adolescents (Additional file 1: Table S3).

## Discussion

Risk factors of age, sex and deprivation showed broadly similar relationships with VR injury admission in WA and England. In both countries, rates increased with deprivation and girls had lower rates than boys except for higher rates in girls in WA in the 11–15 age group. Adjusted incidence rates were similar in the middle age range, but were higher in WA than England at the extremes of age. Several factors may have contributed to these differences. Admission rates for any injury were higher in WA, with rates higher for adolescent girls, and proportionately more injury admissions in adolescents in WA were coded for adverse social circumstances. Among children less than 3 years old, the incidence and proportion of injury admissions coded specifically for maltreatment was higher in WA than in England.

Our findings are consistent with well-established risk factors for victimisation [6,8,30,31]. The steep increase in violence with age and deprivation and its predominance in boys have been reported in community surveys, mortality data, emergency department attendances and hospital admissions [2,6,8,32]. Diminishing adult supervision and increasing time spent outside the home, combined with impulsive or risk taking behaviour and substance abuse, have been identified as risk factors for exposure to violence during adolescence [6,33-35].

The focus of our study was to assess whether there were differences in risk factors and rates between two settings that might reflect potential anomalies in the way victimisation is recognised, recorded or coded in different settings. Such differences could affect the validity of the measure of VR injury for regional comparisons. The excess in adolescent girls in WA has not previously been reported and needs to be examined across other types of injury, such as self-harm and alcohol and drug intoxication. One potential explanation is gender bias in diagnostic coding. For example, 16–17 year old admissions in WA for girls were more likely to have adverse social circumstances codes, whereas boys were most likely to be coded as assault (Additional file 2: Figure S1). Alternatively, there may be a true increase in VR injury admission in young adolescent girls, as suggested by the consistent increase in this age group in both countries.

Variation in coding of sub-categories of VR injury admissions may partly explain the differences in the incidence and proportion of VR injury admissions. The increased rate of admissions coded for adverse social circumstances in 16–17 year olds in WA contributed to a large proportion of the crude rate difference. One possible explanation is increased recognition of social factors in WA in this age group, possibly due to involvement of paediatricians or children’s nurses. In England, most young people in this age group are admitted by adult services, which may pay less attention to social or family factors. Another factor affecting coding sub-categories could be coding requirements. In England, coders are required to record only definite or probable diagnoses or external causes, not causes that are possible or suspected. In WA, all injury admissions are required to have an external cause code [15]. These directives may have affected use of specific codes for maltreatment or assault in all age groups.

Disincentives to record child victimisation include the stigmatising nature and intrinsic uncertainty of the diagnosis [12,17,18,36]. Previous validation studies that compared hospital administrative codes with case notes have shown that clusters of hospital discharge codes for child maltreatment are highly specific but insensitive [11,20,21]. We therefore used a cluster of codes to capture admissions where victimisation was likely to have been considered (eg; codes reflecting undetermined cause or adverse social circumstances) and not just cases where victimisation was specifically recorded (codes reflecting maltreatment or assault). In a validation study against case note review in one hospital in England, we found these codes to be highly specific for victimisation in children admitted for injury (personal communication, Gonzalez-Izquierdo). Quantifying sensitivity is more difficult, but a priori, administrative codes are likely to underestimate the incidence of victimisation-related injury admissions because of failures by clinicians or coders to recognise or record victimisation-related injury [12,17-19].

### Implications

Assessment of the burden of victimisation-related injury is important to inform preventive policies. It is also important to monitor what action is taken after discharge from hospital and whether information is shared with other agencies. Such information could be obtained at low cost through linkage between hospital admission and child protection agency, primary care, education and youth offender databases [13]. For hospital providers, measurement of VR injury is important for ensuring adequate recognition and for costing. Hospital clinicians spend considerable resources liaising with social workers, primary care, mental health or school health services for children who may have been victimised, but this activity is not formally captured in pricing tariffs [37]. Future studies need to assess the association between the VR cluster of ICD-10 codes and clinician consideration of victimisation and how this is reflected in terms of staff time, referrals, and use of effective interventions for this vulnerable group of children and young people [11].

## Conclusion

The consistency in risk factors and rates found in England and WA provides indirect evidence that hospital discharge codes for victimisation-related injury are measuring a similar underlying problem in both settings. The variation in sub-categories highlights the importance of using the whole VR cluster of codes across age and gender groups and different settings. Overall, VR injury admission provides a useful measure for policy and healthcare providers to measure service use and outcomes, although further validation and development of better measures of action taken and resource use is warranted.

## Abbreviations

AIC: Akaike Information Criteria; NICE: National Institute for Health and Care Excellence; VR: Victimisation-related; WA: Western Australia.

## Competing interests

AGI was supported by funding from the Department of Health (Clinical Strategies and Clinical Audit Division) and the Policy Research Programme through funding to the Policy Research Unit in the Health of Children, Young People and Families. The Policy Research Unit in the Health of Children, Young People and Families is funded by the Department of Health Policy Research Programme. This is an independent report commissioned and funded by the Department of Health. The views expressed are not necessarily those of the Department. We would like to thank members of the Policy Research Unit for the health of children, young people and families: Terence Stephenson, Catherine Law, Amanda Edwards, Ruth Gilbert, Steve Morris, Helen Roberts, Catherine Shaw, Russell Viner and Miranda Wolpert. The Centre for Paediatric Epidemiology and Biostatistics was supported in part by the Medical Research Council in its capacity as the MRC Centre of Epidemiology for Child Health. Research at the UCL Institute of Child Health and Great Ormond Street Hospital for Children receives a proportion of the funding from the Department of Health’s National Institute for Health Research Biomedical Research Centres funding scheme. The study sponsors played no part in the design, data analysis and interpretation of this study, the writing of the manuscript, or the decision to submit the paper for publication and the authors’ work was independent of their funders.

## Authors’ contributions

RG, AGI and MOD conceived the paper and the analytic plan. AW wrote the first draft. AGI carried out the analyses with help from MOD and LL, and wrote sections of the paper. RG wrote the final version. All authors commented on the analyses and report. RG is guarantor. All authors read and approved the final manuscript.

## Pre-publication history

The pre-publication history for this paper can be accessed here:

http://www.biomedcentral.com/1472-6963/13/260/prepub

## Supplementary Material

Additional file 1: Table S1 Hierarchy of ICD 10 diagnostic codes used to classify cause of injury related to child victimisation. **Table S2:** Sensitivity analyses: Incidence and prevalence of victimisation in children aged 0–17 years admitted to hospital with head injury in England and Western Australia between 2000 – 2008, inclusive, according to age and cause. **Table S3:** Incidence rate ratios of victimisation-related admission for head injury: multivariable analyses. **Table S4:** Incidence rate ratios for victimisation-related head injury admission: multivariable analyses where interaction with country was significant. Click here for file

Additional file 2: Figure S1 Incidence of victimisation-related injury at the extremes of the age range of study by country and gender: maltreatment-syndrome or assault (*top row*) and undetermined cause or adverse social circumstances (*bottom row*), in early childhood (*left column*) and adolescence (*right column*). Click here for file
