# Supplementary material for: Cross-country comparison of victimisation-related injury admission in children and adolescents in England and Western Australia
Source: BMC Health Serv Res. 2013 Jul 6;13:260. doi: 10.1186/1472-6963-13-260 (PMC3716984; doi:10.1186/1472-6963-13-260)
Supplement: Additional file 1: Table S1 — Hierarchy of ICD 10 diagnostic codes used to classify cause of injury related to child victimisation. Table S2: Sensitivity analyses: Incidence and prevalence of victimisation in children aged 0–17 years admitted to hospital with head injury in England and Western Australia between 2000 – 2008, inclusive, according to age and cause. Table S3: Incidence rate ratios of victimisation-related admission for head injury: multivariable analyses. Table S4: Incidence rate ratios for victimisation-related head injury admission: multivariable analyses where interaction with country was significant. [file 1472-6963-13-260-S1.docx]

**Table S1** Hierarchy of ICD 10 diagnostic codes* used to classify cause of injury related to child victimisation

|  | **Description** | **ICD-10 code** |
| --- | --- | --- |
|  | **1. Maltreatment** |  |
|  | Maltreatment syndromes | T74 |
|  | Perpetrator of neglect and other maltreatment syndromes | Y06, Y07 |
|  | **2. Assault** |  |
|  | Assault by bodily force | Y04, Y05 |
|  | Other types of assault | X85 - Y03, Y08 - Y09 |
|  | **3. Undetermined cause** |  |
|  | Events of undetermined intent | Y10 - Y34 |
|  | Blood -alcohol and blood-drug tests | Z04.0 |
|  | Examination and observation following other inflicted injury | Z04.5 |
|  | Examination and observation for other reasons: request for expert evidence | Z04.8 |
|  | **4. Adverse social circumstances** |  |
|  | Neonatal withdrawal symptoms from maternal use of drugs of addiction | P96.1 |
|  | Problems related to social environment | Z60 |
|  | Problems related to negative life events in childhood | Z61 |
|  | Other problems related to upbringing | Z62 |
|  | Other problems related to primary support group | Z63 |
|  | Problems related to other legal circumstances | Z65.3 |
|  | Problems related to lifestyle (Except Z72.0 Tobacco use) | Z72 |
|  | Problems related to care-provider dependency | Z74 |
|  | Health supervision and care of foundling | Z76.1 |
|  | Health supervision and care of other healthy infant and child | Z76.2 |
|  | Family history of mental and behavioural disorders | Z81 |
|  | Personal history of other mental and behavioural disorders | Z86.5 |
|  | Personal history of other physical trauma | Z91.6 |
|  | Personal history of other specified risk-factors, not elsewhere classified | Z91.8 |

* In England (using HES) codes may be recorded in up to 20 diagnostic fields (14 before 2007 and 7 before 2002) for each hospital episode. In Western Australia (using HMDS) each admission may have up to 21 diagnostic codes and 4 external cause codes recorded. Cause categories above are exclusive and arranged in a descending hierarchy.

**Table S2 Sensitivity analyses:** Incidence and prevalence of victimisation in children aged 0-17 years admitted to hospital with head injury in England and Western Australia between 2000 – 2008, inclusive, according to age and cause.

| Age group (years) | Cause of injury^1^ | Incidence | | | | Prevalence | | | |
| --- | --- | --- | --- | --- | --- | --- | --- | --- | --- |
|  |  | England | | Western Australia | | England | | Western Australia | |
|  |  | IR^2^ | 95%CI | IR | 95%CI | n | (%) | n | (%) |
| <3 | Victimisation^3^ | 26.4 | (25.6, 27.2) | 37.0 | (32.5, 41.5) | 4,223 | (4.3) | 259 | (5.4) |
|  | Maltreatment | 8.2 | (7.8, 8.7) | 14.6 | (11.8, 17.4) | 1,317 | (1.3) | 102 | (2.1) |
|  | Assault | 3.4 | (3.1, 3.6) | 6.0 | (4.2, 7.8) | 536 | (0.5) | 42 | (0.9) |
|  | Uncertain cause | 7.6 | (7.2, 8) | 4.6 | (3, 6.2) | 1,218 | (1.2) | 32 | (0.7) |
|  | Adverse social circumstances | 7.2 | (6.8, 7.6) | 11.9 | (9.3, 14.4) | 1,152 | (1.2) | 83 | (1.7) |
|  | **Total injury admissions** | **615.9** | **(612.1, 619.8)** | **690.6** | **(671.2, 710.1)** | **98,433** | **(100)** | **4830** | **(100)** |
|  | Mid-year population estimate | 15,981,100 | | 699354 | |  | |  | |
| 3 - 10 | Victimisation | 6.0 | (5.8, 6.3) | 7.4 | (6.2, 8.6) | 2,605 | (2.2) | 143 | (2.1) |
|  | Maltreatment | 0.9 | (0.8, 1) | 1.5 | (1, 2) | 392 | (0.3) | 29 | (0.4) |
|  | Assault | 2.0 | (1.9, 2.1) | 2.8 | (2, 3.5) | 859 | (0.7) | 54 | (0.8) |
|  | Uncertain cause | 2.1 | (2, 2.3) | 0.8 | (0.4, 1.2) | 926 | (0.8) | 15 | (0.2) |
|  | Adverse social circumstances | 1.0 | (0.9, 1.1) | 2.3 | (1.6, 3) | 428 | (0.4) | 45 | (0.6) |
|  | **Total injury admissions** | **274.7** | **(273.1, 276.3)** | **358.1** | **(349.6, 366.5)** | **118,797** | **(100)** | **6947** | **(100)** |
|  | Mid-year population estimate | 43,245,100 | | 1940156 | |  | |  | |
| 11 - 15 | Victimisation | 39.9 | (39.2, 40.6) | 37.4 | (34.1, 40.7) | 11,493 | (15) | 481 | (11) |
|  | Maltreatment | 1.3 | (1.2, 1.4) | 1.4 | (0.8, 2) | 379 | (0.5) | 18 | (0.4) |
|  | Assault | 34.2 | (33.5, 34.9) | 30.2 | (27.2, 33.2) | 9,854 | (12.9) | 388 | (8.9) |
|  | Uncertain cause | 2.2 | (2, 2.4) | 0.9 | (0.3, 1.4) | 628 | (0.8) | 11 | (0.3) |
|  | Adverse social circumstances | 2.2 | (2, 2.4) | 5.0 | (3.8, 6.2) | 632 | (0.8) | 64 | (1.5) |
|  | **Total injury admissions** | **266.0** | **(264.1, 267.8)** | **338.7** | **(328.6, 348.7)** | **76,587** | **(100)** | **4356** | **(100)** |
|  | Mid-year population estimate | 28,796,400 | | 1286214 | |  | |  | |
| 16 - 17 | Victimisation | 112.2 | (110.2, 114.1) | 192.3 | (180.4, 204.3) | 13,018 | (35.9) | 1001 | (37.1) |
|  | Maltreatment | 0.5 | (0.3, 0.6) | 3.5 | (1.9, 5.1) | 55 | (0.2) | 18 | (0.7) |
|  | Assault | 106.0 | (104.2, 107.9) | 165.1 | (154, 176.1) | 12,308 | (34) | 859 | (31.8) |
|  | Uncertain cause | 1.9 | (1.7, 2.2) | 1.2 | (0.2, 2.1) | 226 | (0.6) | 6 | (0.2) |
|  | Adverse social circumstances | 3.7 | (3.3, 4) | 22.7 | (18.6, 26.8) | 429 | (1.2) | 118 | (4.4) |
|  | **Total injury admissions** | **312.2** | **(309, 315.4)** | **519.0** | **(499.4, 538.6)** | **36,238** | **(100)** | **2701** | **(100)** |
|  | Mid-year population estimate | 11,607,100 | | 520423 | |  | |  | |

^1^Descending, exclusive hierarchy

^2^Incidence rate per 100,000

^3^Victimisation includes maltreatment, assault, undetermined causes and adverse social circumstances

**Table S3** Incidence rate ratios of victimisation-related admission for head injury: multivariable analyses

| Age group (years) | Factors | Multivariable (no interactions) | |
| --- | --- | --- | --- |
|  |  | IRR | (95%CI) |
| <3 |  |  |  |
|  | Western Australia | 1.01 | (0.86, 1.19) |
|  | Year of age | 0.53 | (0.5, 0.56) |
|  | Female | 0.77 | (0.71, 0.85) |
|  | 2^nd^ least deprived | 1.03 | (0.86, 1.22) |
|  | 3 | 1.57 | (1.33, 1.85) |
|  | 4 | 2.63 | (2.25, 3.07) |
|  | Most deprived | 5.39 | (4.65, 6.25) |
| 3 – 10 |  |  |  |
|  | Western Australia | 0.88 | (0.71, 1.09) |
|  | Year of age | 0.96 | (0.94, 0.99) |
|  | Female | 0.5 | (0.44, 0.57) |
|  | 2^nd^ least deprived | 1.11 | (0.88, 1.4) |
|  | 3 | 1.61 | (1.29, 2) |
|  | 4 | 2.51 | (2.03, 3.09) |
|  | Most deprived | 4.74 | (3.87, 5.8) |
| 11 – 15 |  |  |  |
|  | Western Australia | 0.64 | (0.57, 0.73) |
|  | Year of age | 1.69 | (1.64, 1.74) |
|  | Female | 0.35 | (0.32, 0.37) |
|  | 2^nd^ least deprived | 1.01 | (0.89, 1.15) |
|  | 3 | 1.46 | (1.29, 1.65) |
|  | 4 | 2.29 | (2.03, 2.57) |
|  | Most deprived | 4.28 | (3.82, 4.81) |
| 16 – 17 |  |  |  |
|  | Western Australia | 1.44 | (1.2, 1.72) |
|  | Year of age | 1.26 | (1.06, 1.5) |
|  | Female | 0.18 | (0.15, 0.22) |
|  | 2^nd^ least deprived | 1.08 | (0.82, 1.44) |
|  | 3 | 1.54 | (1.16, 2.05) |
|  | 4 | 1.83 | (1.39, 2.42) |
|  | Most deprived | 3.83 | (2.9, 5.07) |

Overall p-value <0.001 for all models.

**Table S4** Incidence rate ratios for victimisation-related head injury admission: multivariable analyses where interaction with country was significant

| Age group | Factor | England | 95%CI | Western Australia | 95%CI | p-value for interaction w/country** |
| --- | --- | --- | --- | --- | --- | --- |
| 11 – 15* | Female | 0.32 | (0.29, 0.36) | 0.61 | (0.41, 0.92) | <0.001 |
| 16 – 17* | Female | 0.14 | (0.12, 0.17) | 0.30 | (0.18, 0.51) | <0.001 |

* Adjusted by year of age and deprivation

** log (μ) = a + b.WA + c.Deprivation + d.WA.Deprivation + e.Age + f.WA.Age + g.Gender + h.WA.Gender.

Where a, c, e and g are the intercept and coefficients for England, and b, d, f and h are the intercept and coefficients for Western Australia. Deprivation was imputed as numeric (Incidence rate ratio represents increased risk per quintile of deprivation, from least to most deprived)
