# Supplementary material for: Cross-country comparison of victimisation-related injury admission in children and adolescents in England and Western Australia
Source: BMC Health Serv Res. 2013 Jul 6;13:260. doi: 10.1186/1472-6963-13-260 (PMC3716984; doi:10.1186/1472-6963-13-260)
Supplement: Additional file 2: Figure S1 — Incidence of victimisation-related injury at the extremes of the age range of study by country and gender: maltreatment-syndrome or assault (top row) and undetermined cause or adverse social circumstances (bottom row), in early childhood (left column) and adolescence (right column). [file 1472-6963-13-260-S2.docx]

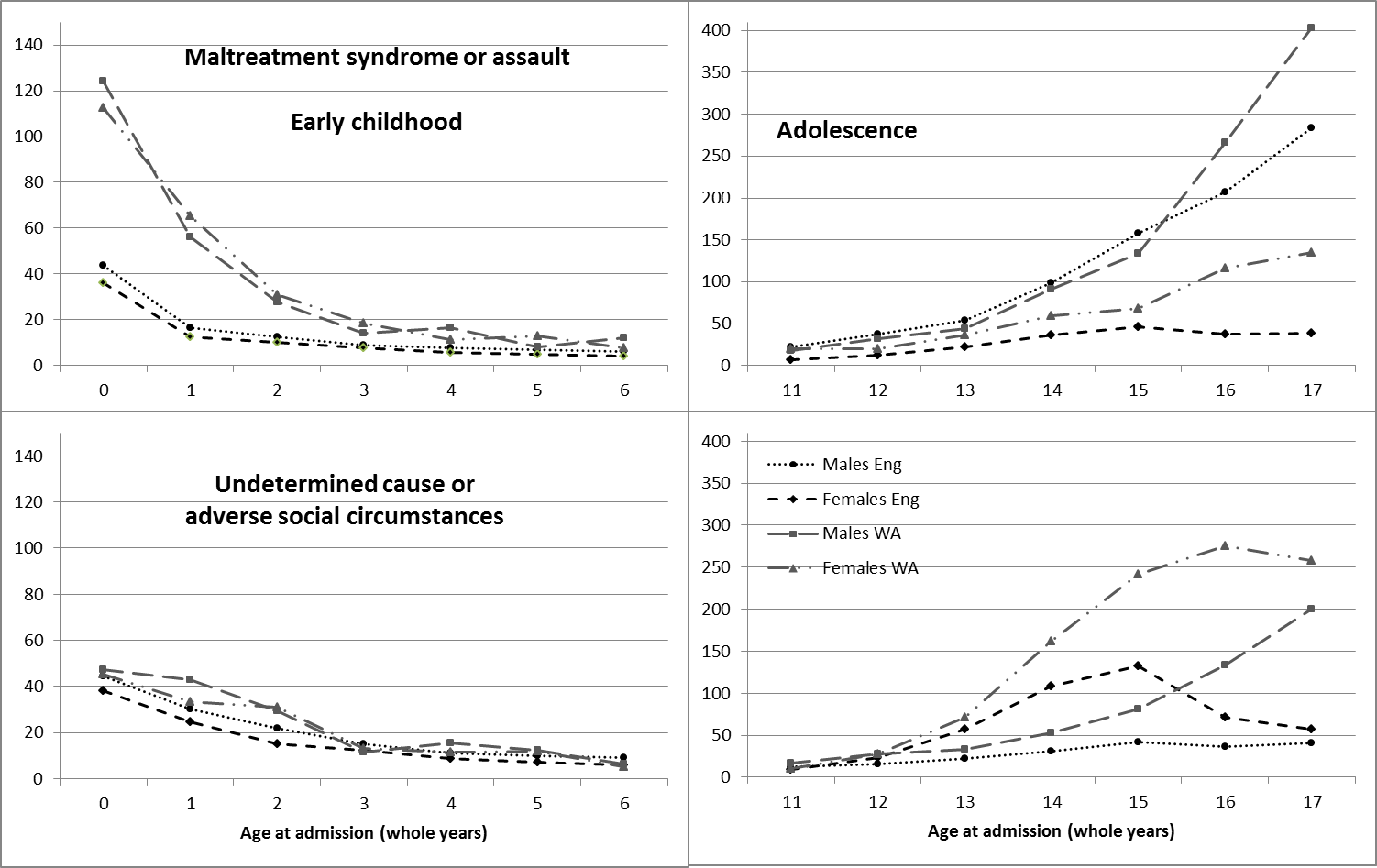


**Figure S1** Incidence of victimisation-related injury at the extremes of the age range of study by country and gender: maltreatment-syndrome or assault (*top row*) and undetermined cause or adverse social circumstances (*bottom row*), in early childhood (*left column*) and adolescence (*right column*)
